# A Case Report of Chilaiditi’s Syndrome With Sigmoid Volvulus

**DOI:** 10.7759/cureus.46193

**Published:** 2023-09-29

**Authors:** Abdul Wahab, Moula Ghulam, Nouman Anthony, Imran Khan, Najeeb Ullah

**Affiliations:** 1 Internal Medicine, Hayatabad Medical Complex Peshawar, Peshawar, PAK; 2 Medicine, Rehman Medical Institute, Peshawar, PAK; 3 General Medicine, Rehman Medical Institute, Peshawar, PAK; 4 Cardiothoracic Surgery, St Bartholomew's Hospital, London, GBR; 5 Internal Medicine, Rehman Medical Institute, Peshawar, PAK

**Keywords:** colostomy reveral, colostomy, sigmoid volvulus, abdominal tuberculosis, chilaiditi’s syndrome

## Abstract

Chilaiditi’s syndrome is the hepatodiaphragmatic interposition of the colon. It can be caused by any pathology of intestinal, hepatic, and diaphragmatic factors. Any anatomic variations or functional abnormalities can increase the development of Chilaiditi’s syndrome. It is usually asymptomatic and is found indecently in radiological studies. It is treated conservatively as long as any complications do not arise. This case of Chilaiditi’s syndrome was associated with sigmoid volvulus and multiple tubercles on its surface.

A 35-year-old male patient presented to the outpatient department (OPD) with complaints of weight loss, bilateral flank pain, abdominal distention, decreased appetite, vomiting, and diarrhea. CT scan showed a grossly distended loop of the colon with sigmoid volvulus and Chilaiditi’s sign. A laparotomy was done, sigmoid volvulus was relieved, a biopsy of tubercles was taken for histopathology, and a colostomy was done. The biopsy result showed abdominal tuberculosis. The colostomy was later reversed.

Chilaiditi’s syndrome is usually treated surgically because it is associated with other complications in the gastrointestinal tract. Previous studies showed the management of cases by colonic resection with primary anastomosis; however, there was one case that reported mortality due to an anastomosis leak.

In this article, we present a case of Chilaiditi’s syndrome associated with sigmoid volvulus and abdominal tuberculosis as seen on biopsy, which was managed surgically by colostomy followed by colostomy reversal on follow-up.

## Introduction

Chilaiditi’s sign is the hepatodiaphragmatic interposition of the colon described by Chilaiditi in 1910 [1]. Chilaiditi’s sign along with clinical symptoms is called Chilaiditi’s syndrome. Chilaiditi’s sign is a rare finding and is seen incidentally on abdominal or chest radiographs with 0.025%-0.28% incidence [2].

The pathogenesis of Chilaiditi’s syndrome is dependent on intestinal, hepatic, and diaphragmatic factors. The interposition of the colon between the diaphragm and liver is prevented by the colon’s fixation and suspensory ligaments, which support it [3]. However, in rare cases, there are anatomical variations that include pathologies such as congenital malposition and suspensory ligament pathologies, which include elongation, laxity, or the complete absence of dolichocolons. Functional disorders can also lead to the development of Chilaiditi’s syndrome including constipation, cirrhosis of the liver, obesity, multiple pregnancies, ascites, aerophagia, diaphragmatic paralysis, and chronic lung disease. Mental abnormalities such as schizophrenia can also result in Chilaiditi’s syndrome [4,5].

In the majority of cases, the condition is asymptomatic and mostly diagnosed on radiological investigations as an incidental finding, but if symptomatic, it shows mostly pathological abdominal signs. Conservative treatment is limited to symptomatic relief only as it cannot change the course of the disease as well as its complications and its recurrence in the future, for which invasive surgical techniques are the best modality of choice as compared to conservative options available even as a preventive measure [6,7].

## Case presentation

A 35-year-old male patient presented to the outpatient department (OPD) with chief complaints of weight loss for the last three months along with bilateral flank pain, abdominal distention, decreased appetite, vomiting, and diarrhea for one and a half months. According to the patient, he was fine three months back after which he had a progressive weight loss of 50 kg over three months. The patient also complained of bilateral flank pain, gradual in onset, colicky in nature, radiating to the back, aggravating with food intake, and relieved with IV analgesics. It was associated with vomiting after every meal, copious in amount, yellowish in color, and mixed with mucus. The vomiting would be relieved after vomiting food contents. Further complaints by the patient were abdominal distention, decreased appetite, and diarrhea. The frequency of diarrhea was three to five episodes per day, which was watery in nature. The patient’s past medical and surgical history was not significant with any known allergies or any regular medications.

**Figure 1:**
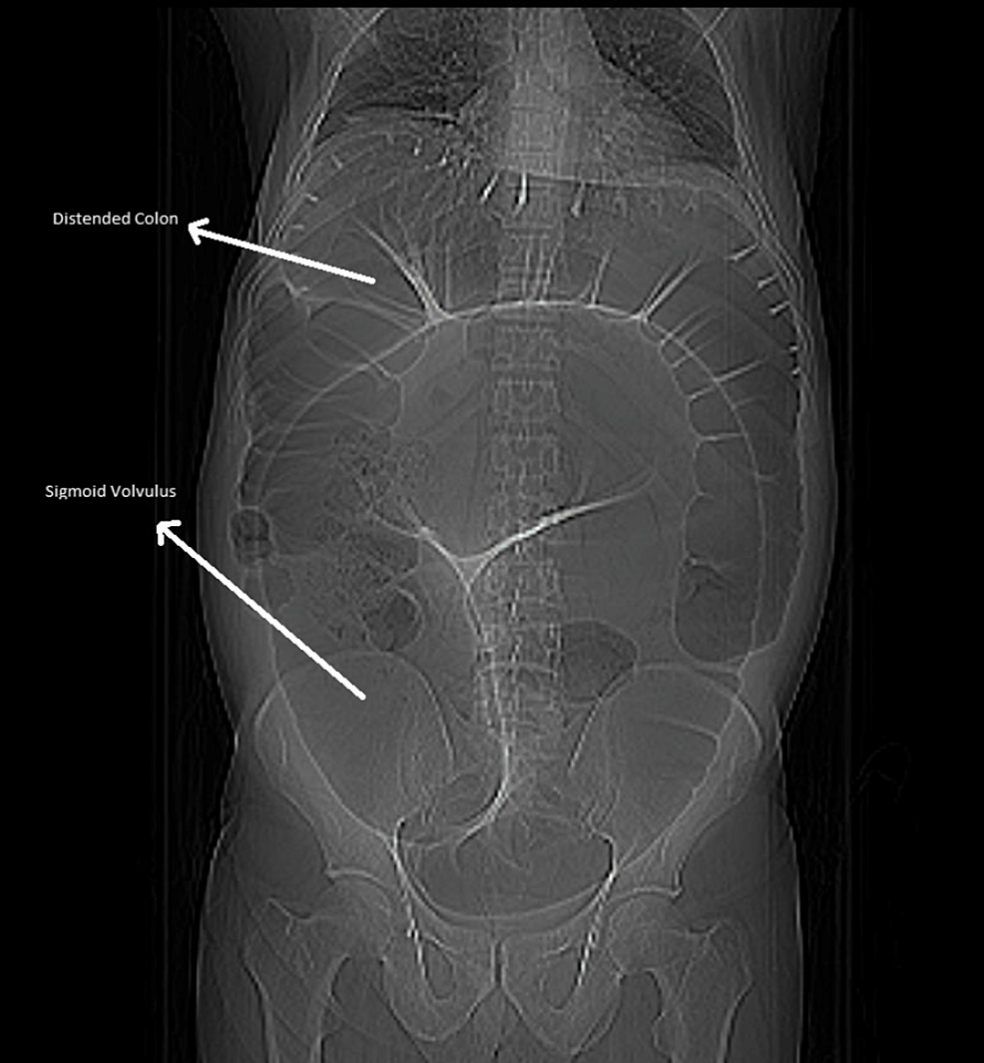
X-ray scan showing a grossly distended loop of the colon with sigmoid volvulus with air under the right side of the diaphragm

On general physical examination, the patient was well-oriented in time, and space. There were no peripheral stigmata with a blood pressure of 130/80 mmHg, pulse of 85 beats per minute, respiratory rate of 20 beats per minute, and oxygen saturation of 98% at room air. Other systemic examinations were unremarkable. On abdominal examination, his abdomen was distended, and there were no visible pulsations, veins, scars, masses, or striations. The abdomen was soft and mildly tender in the left lower quadrant and right upper quadrant. On percussion, the abdomen was dull with positive fluid thrill. Bowel sounds were absent on auscultation. On digital rectal examination, the rectum was empty. Baseline laboratory investigations, such as complete blood count (CBC), urine routine/examination (RE), hepatitis B surface antigen (HBsAg), hepatitis C virus (HCV) Ag, and blood culture, were normal. Liver function tests also showed no significant findings. However, serum electrolytes revealed hyponatremia (122 mEq/L), hypokalemia (2.37 mEq/L), and hypochloremia (90.9 mEq/L). On radiological investigations, an x-ray abdomen was done, which showed sigmoid volvulus with severe dilation of the sigmoid and transverse colon. The descending colon was also dilated, and the rectum collapsed as seen in Figure 1.

**Figure 2:**
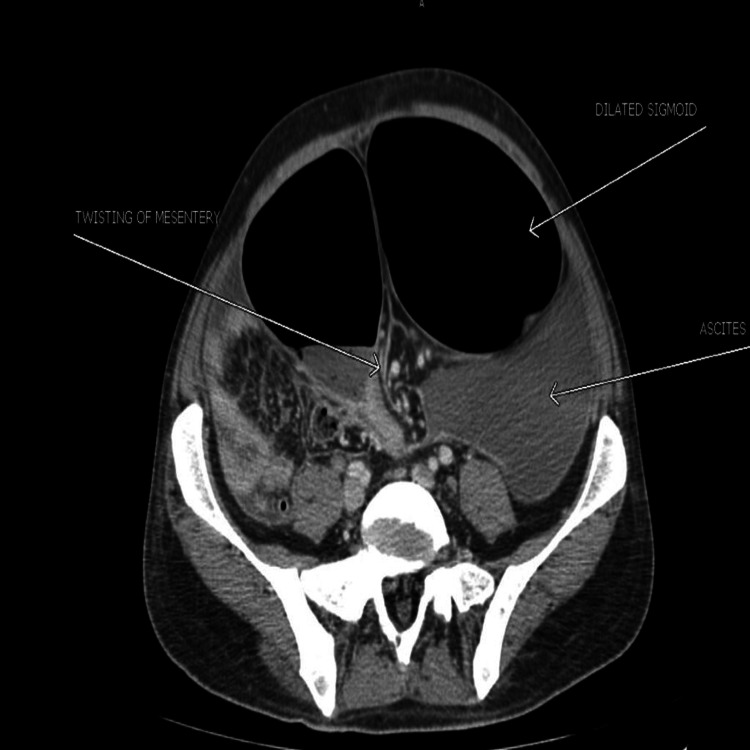
CT scan showing the dilated sigmoid with twisting of the mesentery and gross ascites

On CT liver dynamic study (covering chest) axial view, there was a severely distended sigmoid colon (13.5 cm) and transverse colon (8 cm) with twisting of mesentery with the whirling of mesenteric vessels, which suggests sigmoid volvulus. Mesenteric vessels were patent. There were also gross ascites with diffuse peritoneal thickening and omental nodularity as seen in Figure 2.

**Figure 3:**
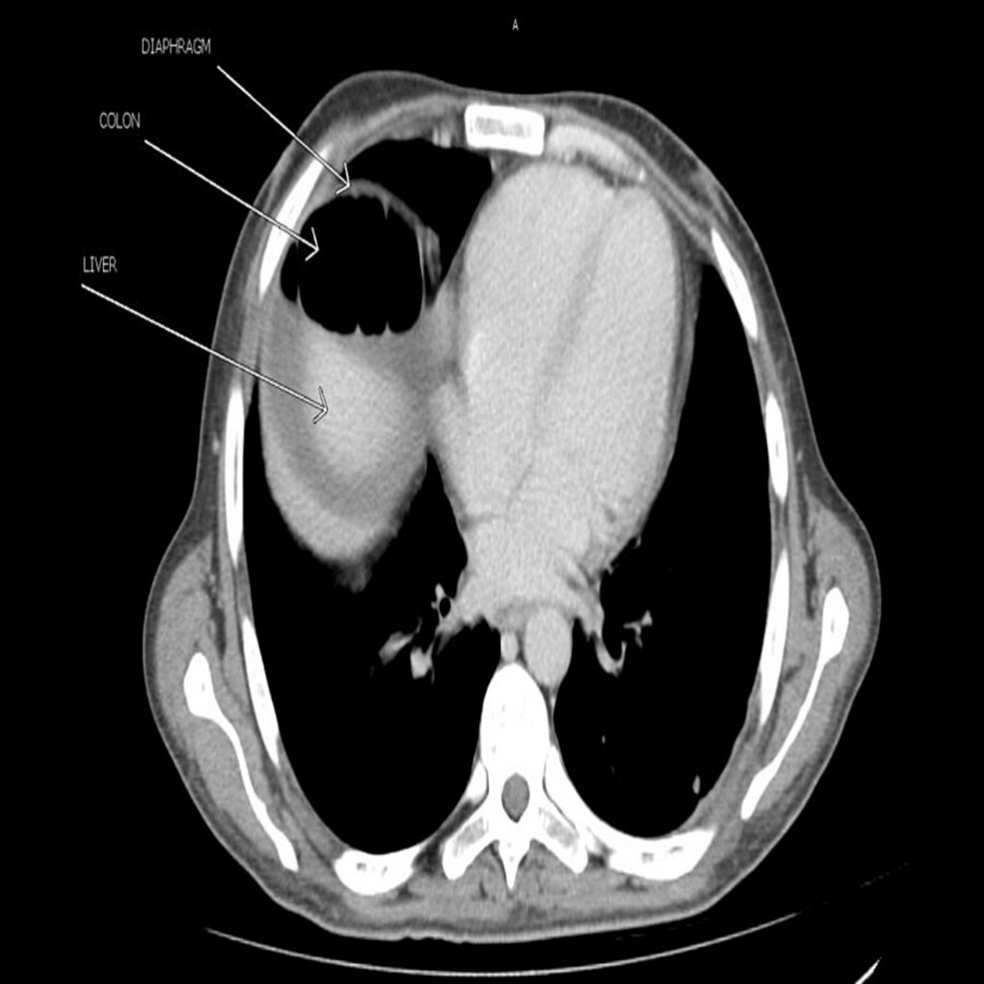
CT scan showing the interposition of the colon between the liver and diaphragm

Another section of the CT scan axial view (Figure 3) showed part of the large bowel in the right subphrenic region. No focal lesion was seen in the liver, spleen, kidneys, pancreas, gall bladder, and adrenals.

There were a few tiny subcentimeter bilateral renal calculi. There were no large para-aortic lymph nodes. No obvious osseous lesions were seen. CT chest revealed few atelectasis bands in both lower lobes. A small pleural effusion was also seen.

A diagnosis of sigmoid volvulus was made for which laparotomy was done. On exploration, the sigmoid volvulus was examined, and multiple tubercles were present throughout the abdomen for which a biopsy was taken. The sigmoid colon was untwisted, and part of it was resected because it was ischemic and had adhesions. A double-barrel colostomy was done. A 6-week follow-up was advised to the patient. Biopsy results revealed abdominal tuberculosis for which anti-TB drugs were prescribed.

The patient came for his follow-up after six weeks for his colostomy reversal. Medications were provided at home, and advice was given on how to properly dress the wound, mobilize the patient, avoid heavy lifting, and consume a healthy diet.

## Discussion

The patient in our study had Chilaiditi’s syndrome with sigmoid volvulus, but multiple tubercles were also seen on the sigmoid colon. A colostomy was done, and a biopsy was taken. The patient was counseled afterward, and a follow-up was suggested when the biopsy report was available. As seen in this study, the mainstay treatment of the condition was surgery. Many cases have been reported globally that are in accordance with the treatment provided in this case [8-10].

Previous studies conducted by separate researchers, such as Williams et al., on Chilaiditi’s syndrome with colonic volvulus have shown that the treatment repeatedly given was partial colonic resection, followed by a primary anastomosis, of which one of them reported mortality because of anastomosis leak [11].

A case report study by Erdem et al. reported a patient with the chief complaint of shortness of breath. The patient in our study did not have this chief complaint [12]. The presence of right subphrenic airspace on a chest X-ray in Chilaiditi’s syndrome has many differential diagnoses, such as subdiaphragmatic abscess, pneumoperitoneum, and diaphragmatic hernia [13]. Chilaiditi’s syndrome can cause numerous complications, including volvulus of the cecum, splenic flexure, transverse and sigmoid colon, cecal perforation, and subdiaphragmatic appendicitis perforation. Undiagnosed Chilaiditi’s sign can increase the risk of colonic perforation during the procedure of colonoscopy and liver biopsy [14].

Chilaiditi’s syndrome can be managed conservatively on its own. Therefore, a thorough complete radiological workup must be done to exclude other differential diagnoses and prevent unnecessary intervention where it is not needed. Kamiyoshihara et al. presented a case of a 75-year-old patient involved in a road traffic accident who was misdiagnosed with a diaphragmatic hernia. When an explorative laparotomy was performed, it turned out to be a Chilaiditi’s syndrome case, which could have been managed conservatively [15].

## Conclusions

We presented a rare case of sigmoid volvulus with multiple tubercles present on its surface in an adult with Chilaiditi’s syndrome. We conclude with surgical correction of the sigmoid volvulus along with a biopsy of tubercles followed by colostomy and secondary anastomosis after the arrival of biopsy results. In the absence of volvulus or ischemia of the colon, Chilaiditi’s syndrome should be managed conservatively.
